# Secukinumab provides sustained PASDAS-defined remission in psoriatic arthritis and improves health-related quality of life in patients achieving remission: 2-year results from the phase III FUTURE 2 study

**DOI:** 10.1186/s13075-018-1773-y

**Published:** 2018-12-07

**Authors:** L. C. Coates, D. D. Gladman, P. Nash, O. FitzGerald, A. Kavanaugh, T. K. Kvien, L. Gossec, V. Strand, L. Rasouliyan, L. Pricop, K. Ding, S. M. Jugl, C. Gaillez, on behalf of the FUTURE 2 study group

**Affiliations:** 1 Nuffield Department of Orthopaedics, Rheumatology and Musculoskeletal Sciences, University of Oxford, Botnar Research Centre, Windmill Road, Oxford, OX3 7LD UK; 2 Department of Medicine, Rheumatology, University of Toronto, Toronto Western Hospital, Toronto, Ontario Canada; 3 Department of Medicine, University of Queensland, Brisbane, Australia; 4 Department of Rheumatology, St Vincent’s University Hospital and Conway Institute for Biomolecular Research, University College Dublin, Dublin, Ireland; 5 UC San Diego School of Medicine, La Jolla, CA USA; 6 Department of Rheumatology, Diakonhjemmet Hospital, Oslo, Norway; 7 Sorbonne Universités, UPMC Université Paris 06, Paris, France; 8 Rheumatology Department, Hôpital Pitié Salpêtrière, AP-HP, Paris, France; 9 Division of Immunology/Rheumatology, Stanford University School of Medicine, Palo Alto, CA USA; 10 RTI Health Solutions, Barcelona, Spain; 11 Novartis Pharmaceuticals Corporation, East Hanover, NJ USA; 12 Novartis Pharma AG, Basel, Switzerland

**Keywords:** Psoriatic arthritis, Secukinumab, PASDAS, Remission, Interleukin-17A, FUTURE 2 study

## Abstract

**Background:**

Secukinumab has demonstrated sustained improvement in the signs and symptoms of psoriatic arthritis (PsA) over 2 years in the FUTURE 2 study (NCT01752634). This post hoc analysis assessed the ability of secukinumab to achieve Psoriatic Arthritis Disease Activity Score (PASDAS)-based remission or low disease activity (LDA) through 2 years among patients with PsA in the FUTURE 2 study.

**Methods:**

PASDAS (cut-off scores: remission ≤ 1.9; LDA > 1.9 and < 3.2; Moderate Disease Activity ≥ 3.2 and < 5.4; and high disease activity [HDA] ≥ 5.4) was assessed in the overall population (tumour necrosis factor inhibitor [TNFi]-naïve and TNFi-experienced), in patients stratified by prior TNFi use and by disease duration at weeks 16, 52 and 104. The impact of secukinumab on individual PASDAS core components and on the relationship between PASDAS states and patient-reported outcomes (PROs), including physical function, health-related quality of life (HRQoL) and work productivity, were also assessed. Data for the approved doses of secukinumab (300 and 150 mg) are reported. PASDAS scores and core components were reported as observed, and PROs were analysed using mixed models for repeated measures.

**Results:**

In the overall population, PASDAS remission and LDA were achieved in 15.6% and 22.9%, respectively, of patients treated with secukinumab 300 mg and in 15.2% and 19.2%, respectively, in the secukinumab 150 mg group versus 2.3% and 13.8%, respectively, with placebo at week 16. In the TNFi-naïve group, a higher proportion of patients achieved remission + LDA at week 16 with secukinumab 300 and 150 mg (46.2% and 42.9%, respectively) versus placebo (17.5%), with corresponding responses in TNFi-experienced patients being 22.6% and 19.4% versus 13.3%. Remission/LDA responses with secukinumab were sustained through 2 years. Patients achieving remission/LDA reported greater improvements in PROs than patients in HDA through 2 years.

**Conclusions:**

Secukinumab-treated patients achieved higher PASDAS-defined remissions or LDA compared with placebo at week 16, which were sustained through 2 years. Remission/LDA was achieved by both TNFi-naïve and TNFi-experienced patients treated with secukinumab, with higher rates in TNFi-naïve patients. Secukinumab-treated patients achieving remission/LDA reported significantly greater improvements in PROs, including physical function and different dimensions of health-related quality of life and work, than patients in HDA.

**Trial registration:**

ClinicalTrials.gov, NCT01752634. Registered on December 19, 2012.

EUDRACT, 2012-004439-22. Registered on December 12, 2012.

## Introduction

Psoriatic arthritis (PsA) is an inflammatory musculoskeletal disease comprising several domains, including peripheral arthritis, axial disease, enthesitis, dactylitis, and skin and nail disease [1, 2]. Different tools, such as Disease Activity Score (DAS) and American College of Rheumatology (ACR) response criteria, which primarily focus on peripheral joint manifestations and were validated in patients with rheumatoid arthritis, have been used for measuring joint responses in PsA. However, outcome measures borrowed from rheumatoid arthritis, such as ACR response criteria and DAS28-CRP, do not reflect the variety of disease manifestations [3, 4]. One of the overarching principles of the treat-to-target recommendations for patients with PsA by international task forces [5], the Group for Research and Assessment of Psoriasis and Psoriatic Arthritis (GRAPPA) [6], and the European League Against Rheumatism (EULAR) [7] is to achieve the lowest possible level of disease activity across all domains of disease. Disease remission and low/minimal disease activity are defined by the minimal disease activity (MDA), which measures all domains of the disease, or Disease Activity Index for Psoriatic Arthritis (DAPSA), which measures peripheral arthritis and acute-phase reactants [4, 8, 9].

Another measure, the Psoriatic Arthritis Disease Activity Score (PASDAS), was developed by the GRAPPA Composite Exercise Project and provides different validated thresholds to identify remission, low disease activity (LDA), moderate disease activity (MoDA) and high disease activity (HDA) [10]. PASDAS assesses multiple manifestations of PsA, distinguishes treatment effects, performs better than traditional joint-only indices, and can be used as a treatment target in PsA randomized controlled trials (RCTs) [10–12].

Secukinumab, a fully human IgG1 monoclonal antibody that selectively neutralizes IL-17A, has substantial efficacy in the treatment of moderate to severe psoriasis, PsA and ankylosing spondylitis, demonstrating rapid onset of action and sustained responses with a favourable safety profile [13–19]. In the placebo-controlled, double-blind, phase III FUTURE 2 trial (NCT01752634), secukinumab treatment resulted in significant improvements in key clinical domains of PsA compared with placebo, and these improvements were sustained through week 104 [15, 16].

In the present study, we explored the ability of secukinumab-treated patients to achieve remission or LDA using PASDAS scores at weeks 16, 52 and 104 in the FUTURE 2 study. This post hoc analysis also determined the impact of secukinumab on individual components of PASDAS and the relationship of PASDAS states with patient-reported outcomes (PROs), including health related-quality of life (HRQoL), physical function, work productivity and activity impairments.

## Methods

### Study design and patients

FUTURE 2 is a phase III multicentre RCT designed to evaluate the efficacy and safety of subcutaneous secukinumab treatment in patients with active PsA. Details of the study design, inclusion and exclusion criteria, and 104-week efficacy and safety results have been reported previously [15, 16]. Briefly, patients were randomized (1:1:1:1) to receive subcutaneous (s.c.) secukinumab 300, 150 or 75 mg or placebo at baseline; weeks 1, 2, 3 and 4; and every 4 weeks thereafter. Placebo-treated patients were re-randomized to receive secukinumab 300 or 150 mg at either week 16 or week 24, based on clinical responses [15]. Data only with the approved doses of secukinumab (i.e., 300 and 150 mg) and placebo are reported herein.

The study was conducted in accordance with the principles of the Declaration of Helsinki, the International Conference on Harmonisation good clinical practice guidelines, and all applicable laws and regulations. All centres received approval from independent ethics committees or institutional review boards (IRBs). Patients provided written informed consent before starting the study-related procedures. The details of the study were mentioned in the earlier FUTURE 2 publications [15, 16].

### Outcomes

PASDAS is a composite score that includes physician and patient global assessment of skin and joint disease by visual analogue scale (VAS), the physical component summary score of the Medical Outcomes Study Short Form Health Survey (SF-36 PCS), swollen joint count based on 66 joints (SJC66), tender joint count based on 68 joints (TJC68), Leeds Enthesitis Index enthesitis count, tender dactylitis count and CRP level [6, 11]. The continuous PASDAS score [11] was calculated for each patient with evaluable data at each point of interest. Disease activity states are defined as follows:

- Remission: PASDAS score ≤ 1.9
- LDA: 1.9 < PASDAS score < 3.2
- MoDA: 3.2 ≤ PASDAS score < 5.4
- HDA: PASDAS score ≥ 5.4

The proportions of patients in remission, LDA, MoDA and HDA were assessed at weeks 16, 52 and 104 in the overall population, and patients stratified by prior TNF inhibitor treatment (TNFi-naïve or TNFi-experienced) or by time since diagnosis (≤ 2 versus > 2 years). Shifts in disease activity in patients in each PASDAS state at week 16 were evaluated for secukinumab 300 and 150 mg treatment to assess sustainability of responses at weeks 52 and 104.

### PASDAS states and individual core components

The impact of secukinumab treatment on individual PASDAS components was calculated among patients categorized in each disease activity state at weeks 16, 52 and 104.

### PASDAS states and patient-reported outcomes

The relationship between PASDAS disease activity and PROs was assessed at weeks 16, 52 and 104 using data pooled across the treatment groups. HRQoL was assessed using SF-36 PCS (a component of the PASDAS score) and SF-36 MCS scores, Psoriatic Arthritis Quality of Life questionnaire (PsAQoL), Dermatology Life Quality Index (DLQI), physical function by Health Assessment Questionnaire Disability Index (HAQ-DI), fatigue by Functional Assessment of Chronic Illness Therapy–Fatigue (FACIT-Fatigue) and work productivity by the Work Productivity and Activity Impairment (WPAI) questionnaire.

### Statistical analysis

The percentages of patients in each PASDAS disease activity state in the overall population (TNFi-naïve and TNFi-experienced) and by prior TNFi treatment status and disease duration at baseline were reported using mutually exclusive categories at the group level according to as-observed analyses, including shifts in disease activity states from week 16 to weeks 52/104.

Median, first (Q1) and third quartiles (Q3) were computed for each of the individual core PASDAS components at weeks 16, 52 and 104 for each disease activity state in patients with data at weeks 16/52 and 16/104.

Additionally, SF-36 PCS and MCS scores, PsAQoL, DLQI, HAQ-DI, FACIT-Fatigue and WPAI were assessed at weeks 16, 52 and 104 using mixed models for repeated measures (MMRM) analyses with analysis visit, PASDAS disease activity state at the analysis visit (remission, LDA, MoDA and HDA), and randomization stratum (TNFi-naïve or TNFi-experienced) as factors and weight and baseline score as continuous covariates. PASDAS disease activity state and baseline score by analysis visit were included as interaction terms. An unstructured covariance structure was used for MMRM analysis. Least-squares mean (LSM) estimates and SEs along with *P* values were provided at each assessment for patients in each disease activity state. Analysis data were pooled across treatment arms (secukinumab + placebo) pertaining to relationship between PASDAS disease activity states and PROs. All statistical analyses were performed using SAS version 9.4 or higher software (SAS Institute, Cary, NC, USA).

## Results

### Patients

Demographic and baseline characteristics were well balanced across treatment groups in FUTURE 2 [15]; the parameters relevant to PASDAS are shown in Table 1. Mean (SD) PASDAS scores at baseline were 5.9 (0.9), 6.0 (1.0) and 5.8 (1.0) in the secukinumab 300 mg, secukinumab 150 mg and placebo groups, respectively. At baseline, > 60% of patients were TNFi-naïve across the groups (Table 1). Retention rates of enrolled patients at week 104 were 86% (86/100) and 76% (76/100) with secukinumab 300 and 150 mg, respectively. Discontinuations due to lack of efficacy at week 104 were 3% (3/100) and 7% (7/100) in the secukinumab 300 and 150 mg groups, respectively [16].

**Table 1:** Demographics and baseline disease characteristics relevant to Psoriatic Arthritis Disease Activity Score

Characteristic mean (SD) unless otherwise stated	Secukinumab 300 mg s.c. (*N* = 100)	Secukinumab 150 mg s.c. (*N* = 100)	Placebo (*N* = 98)
Age, years	46.9 (12.6)	46.5 (11.7)	49.9 (12.5)
Female, *n* (%)	49 (49.0)	45 (45.0)	59 (60.2)
Time since diagnosis of PsA in years	7.4 (7.5)	6.5 (8.2)	7.3 (7.8)
TNFi-naïve, *n* (%)	67 (67.0)	63 (63.0)	63 (64.3)
Psoriasis ≥ 3% of BSA, *n* (%)	41 (41.0)	58 (58.0)	43 (43.9)
Physician’s global VAS	55.0 (14.7)	56.7 (16.6)	55.0 (16.0)
Patient’s global VAS	60.7 (18.9)	62.0 (19.5)	57.6 (19.8)
SF-36 PCS	36.9 (8.0)	36.2 (8.1)	37.4 (8.8)
Dactylitis count^a^	3.6 (3.5)	4.5 (5.1)	2.7 (2.2)
Enthesitis count^b^	2.8 (1.7)	3.2 (16)	3.1 (1.7)
TJC (78 joints)	20.2 (13.3)	24.1 (19.4)	23.4 (19.0)
SJC (76 joints)	11.2 (7.8)	11.9 (10.1)	12.1 (10.7)
PASDAS score	5.9 (0.9); n = 98	6.0 (1.0); *n* = 99	5.8 (1.0); *n* = 98

*Abbreviations: BSA* Body surface area, *SJC* Swollen joint count, *TJC* Tender joint count, *VAS* Visual analogue scale

n, number of patients in each treatment group providing data; N, number of randomized patient

^a^The dactylitis count is the number of fingers and toes with dactylitis, with a range of 0–20 and if dactylitis is present with any finger or toe, the patient is counted as a patient with dactylitis

^b^Enthesitis was evaluated by Leeds Enthesitis Index, a validated enthesitis index. If enthesitis is present in any of the 6 sites (lateral epicondyle humerus L + R, proximal Achilles L + R and medial condyle femur L + R), the patient is counted as a patient with enthesitis

### PASDAS states

**Fig. 1:**
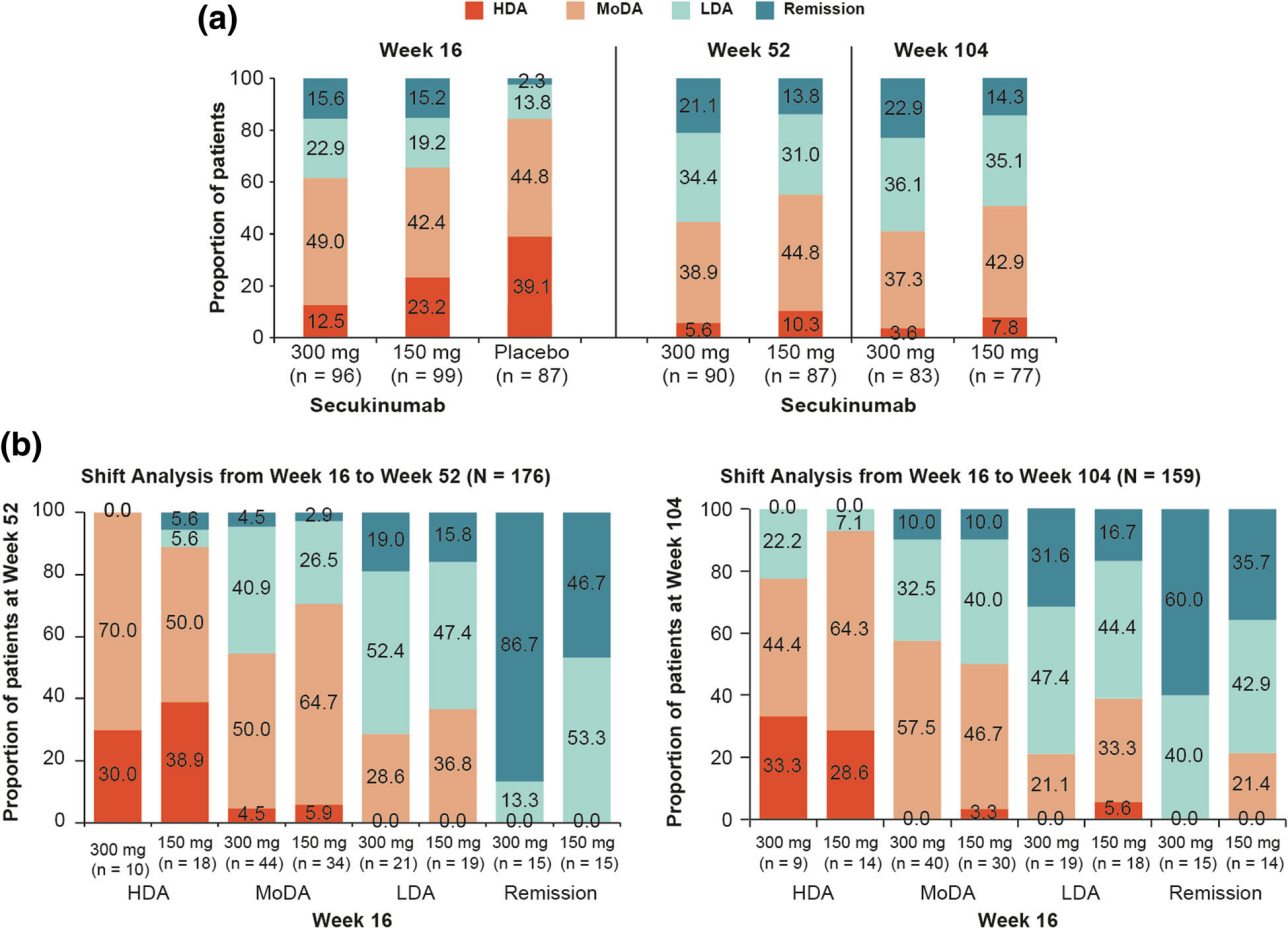
**a** Proportion of patients achieving Psoriatic Arthritis Disease Activity Score (PASDAS) remission, LDA, MoDA and HDA at weeks 16, 52 and 104 in overall population. Data were reported using mutually exclusive categories at group level and as observed analysis. LDA: 1.9 < PASDAS score < 3.2; remission: PASDAS score ≤ 1.9. Secukinumab 300 and 150 mg data are reported (approved doses). *n* = number of patients in the treatment group with evaluation. **b** Shift analysis of PASDAS disease activity states from week 16 to week 52 or 104. Data were reported using mutually exclusive categories at group level and as observed analysis. *n* = number of patients in each PASDAS state at week 16; *N* = total number of patients with non-missing PASDAS scores at week 16 and weeks 52/104. *HDA* High disease activity, *LDA* Low disease activity, *MoDA* Moderate disease activity

In the overall population, a higher proportion of secukinumab 300 mg (38.5% [37/96]) and 150 mg (34.3% [34/99])-treated patients achieved PASDAS remission + LDA at week 16 versus placebo (16.1% [14/87]). At week 104, 22.9% (19/83) and 14.3% (11/77) patients achieved remission, and 36.1% (30/83) and 35.1% (27/77) patients reached LDA with secukinumab 300 and 150 mg, respectively. The proportions of patients in PASDAS remission, LDA, MoDA and HDA at weeks 16, 52 and 104 are depicted in Fig. 1a. The shift analysis from weeks 16 to 104 showed that the majority of secukinumab 300 mg-treated patients who achieved remission at week 16 maintained remission (60%) or were in LDA (40%) at week 104, and 79% of patients in LDA at week 16 maintained or improved their status to remission at week 104; all patients in MoDA at week 16 either maintained or improved their status. In contrast, patients treated with secukinumab 150 mg who achieved remission at week 16 maintained remission (35.7%) or were in LDA (42.9%) or MoDA (22%) at week 104, patients in LDA at week 16 maintained or improved their status (60%) or lost their status (40%), and 97% of patients in MoDA maintained or improved their status at week 104 (Fig. 1b).

**Fig. 2:**
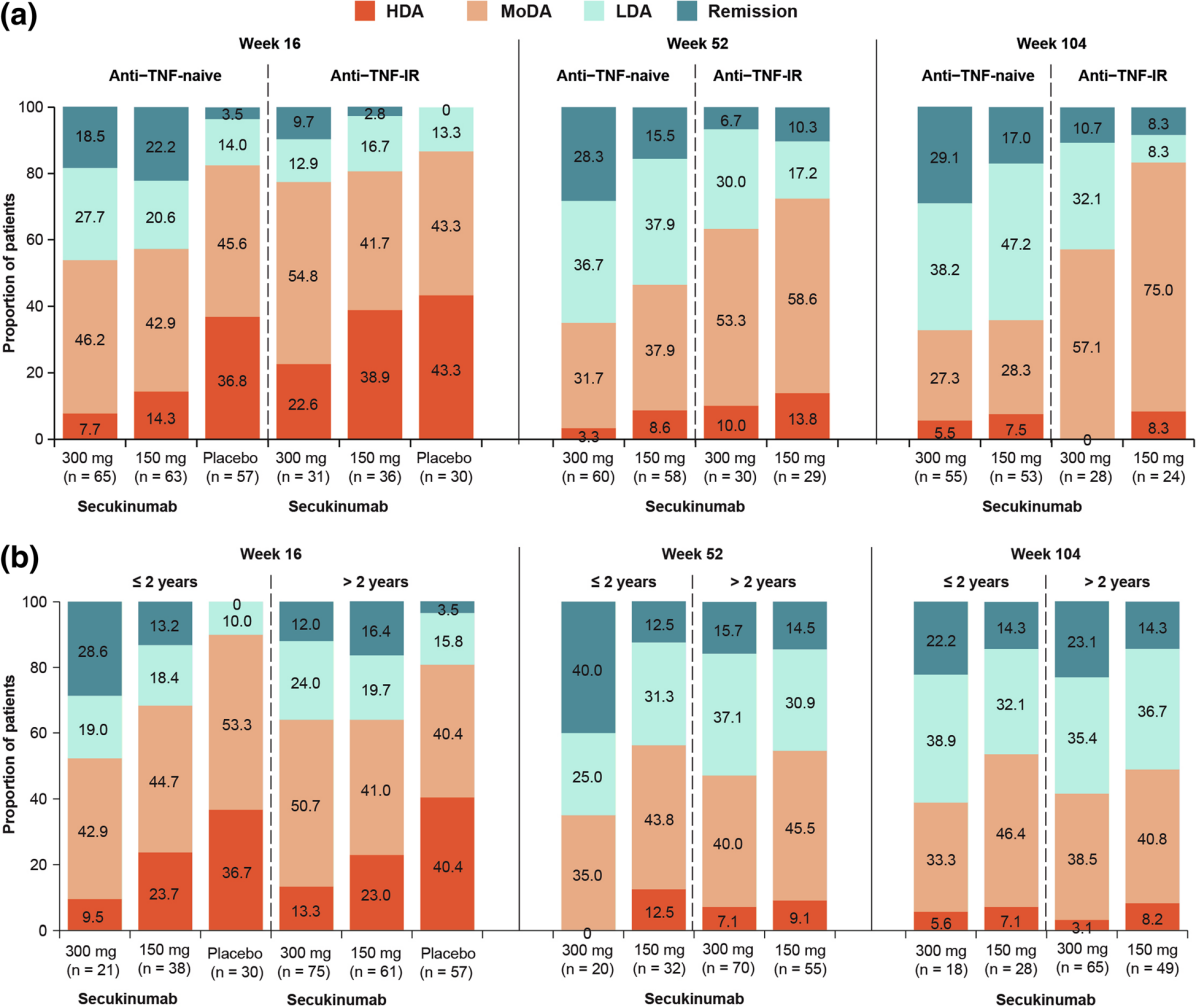
Proportion of patients achieving Psoriatic Arthritis Disease Activity Score (PASDAS) remission, LDA, MoDA and HDA at weeks 16, 52 and 104 by (**a**) Tumour necrosis factor inhibitor (TNFi) status and (**b**) time since psoriatic arthritis diagnosis. *HDA* High disease activity, *LDA* Low disease activity, *MoDA* Moderate disease activity. *n* = number of patients in the treatment group with evaluation

A higher proportion of TNFi-naïve and TNF-experienced patients achieved remission or LDA at week 16 versus placebo with generally higher response rates in the TNFi-naïve subgroup. In TNFi-naïve patients, remission + LDA was achieved by 46.2% (30/65) patients with secukinumab 300 mg and 42.9% (27/63) with 150 mg versus placebo (17.5% [10/57]), with responses in TNFi-experienced patients being 22.6% (7/31) for secukinumab 300 mg and 19.4% (7/36) for 150 mg versus 13.3% (4/30) for placebo. These response rates were sustained at weeks 52 and 104 in both TNFi-naïve and TNFi-experienced subgroups (Fig. 2a). Regardless of time since PsA diagnosis (≤ 2 years versus > 2 years), a higher proportion of secukinumab-treated patients achieved PASDAS remission or LDA than placebo at week 16. This proportion of patients achieving remission/LDA increased further at weeks 52 and 104, regardless of time since PsA diagnosis (Fig. 2b).

### PASDAS states and core components

**Fig. 3:**
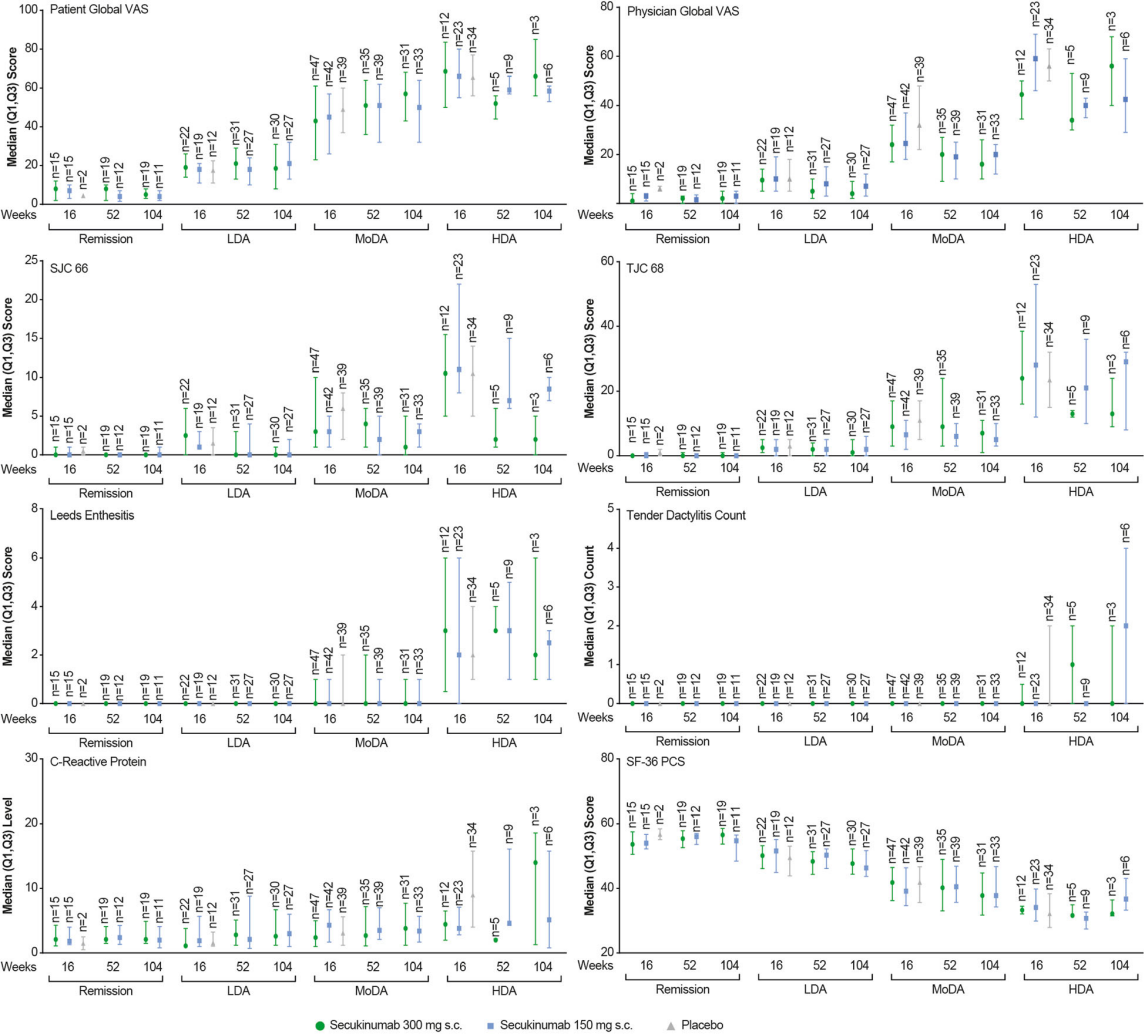
Scores of Psoriatic Arthritis Disease Activity Score (PASDAS) components at weeks 16, 52 and 104. The median value is denoted by symbol in the figure, and the upper and lower error bars represent third (Q3) and first (Q1) quartiles, respectively. *n* = number of patients in respective disease states at assessment. *HDA* High disease activity, *LDA* Low disease activity, *MoDA* Moderate disease activity, *SF-36 MCS* Short Form Health Survey Mental Component Summary, *SJC* Swollen joint count, *TJC* Tender joint count, *VAS* Visual analogue scale

The median (Q1, Q3) scores of PASDAS core components in each PASDAS state at weeks 16, 52 and 104 are shown in Fig. 3. The most improved individual components in patients achieving PASDAS remission and LDA were dactylitis, enthesitis, SF-36 PCS, physician global VAS and SJC at weeks 16, 52 and 104. For dactylitis and enthesitis core components, median improvements were numerically similar in patients reaching PASDAS remission and LDA.

### PASDAS states and PROs

**Fig. 4:**
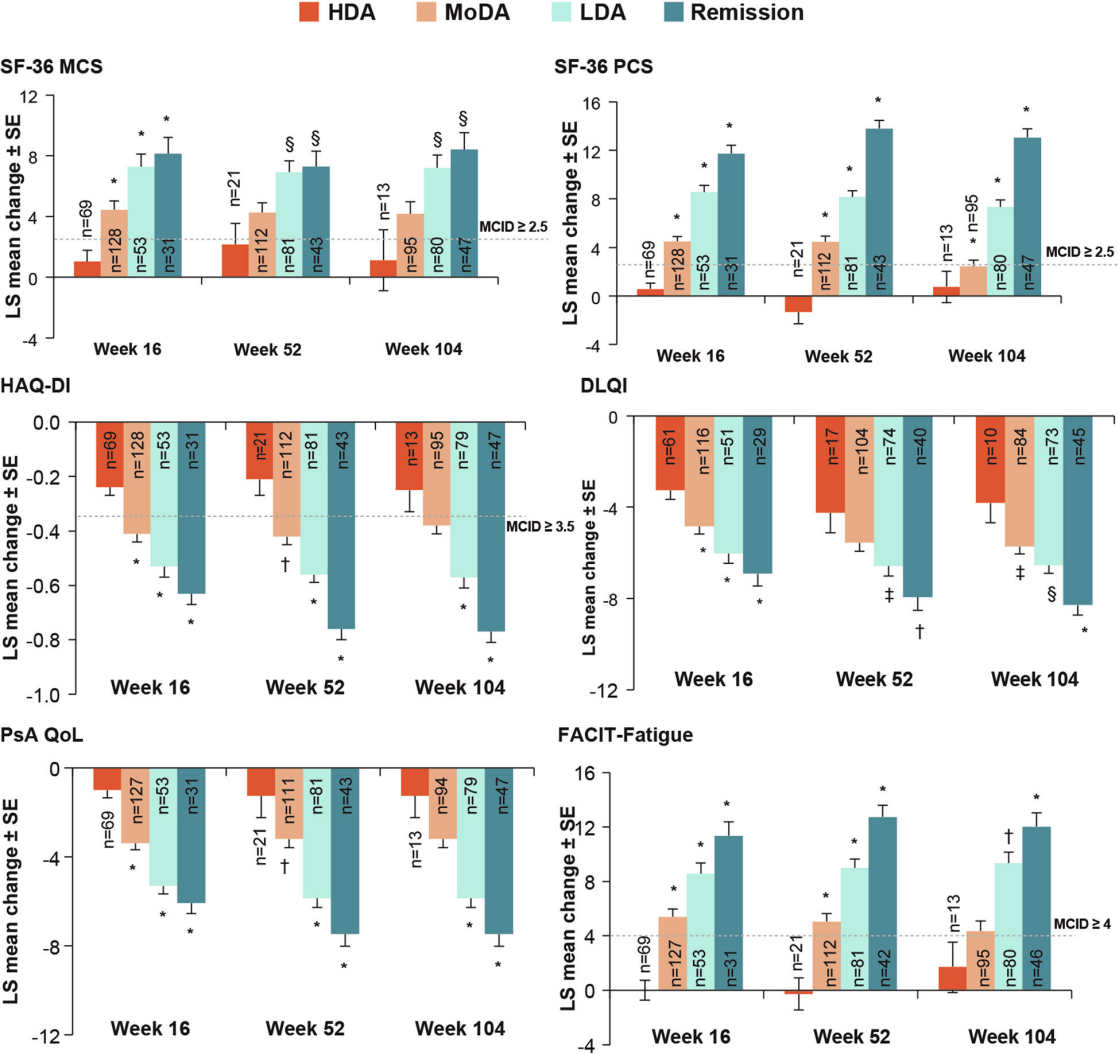
Mean change in patient-reported outcome scores by Psoriatic Arthritis Disease Activity Score (PASDAS) states at weeks 16, 52 and 104. Data are from mixed model for repeated measures analysis. *P < 0.0001; ^†^*P* < 0.001; ^§^*P* < 0.01; ^‡^*P* < 0.05 versus HDA. *n* = number of patients with measurements at baseline and post-baseline visits; *N* = number of patients in each group of the specified analysis set. *DLQI* Dermatology Life Quality Index, *FACIT-Fatigue* Functional Assessment of Chronic Illness Therapy–Fatigue, *HAQ-DI* Health Assessment Questionnaire Disability Index, *HDA* High disease activity, *LDA* Low disease activity, *LS* Least squares, *PsAQoL* Psoriatic arthritis-specific quality of life, *SF-36 MCS* Short Form Health Survey Mental Component Summary, *SF-36 PCS* Short Form Health Survey Physical Component Summary. Week 16: *N* = 32 (remission), *N* = 53 (LDA), *N* = 128 (MoDA) and *N* = 69 (HDA); week 52: *N* = 44 (remission), *N* = 81 (LDA), *N* = 112 (MoDA) and *N* = 21 (HDA); week 104: *N* = 47 (remission), N = 81 (LDA), *N* = 95 (MoDA) and *N* = 13 (HDA). Analytic data were pooled across treatment arms

At week 16, LSM changes from baseline in SF-36 PCS and MCS, PsAQoL, DLQI, HAQ-DI and FACIT-Fatigue scores were significantly higher (*P* < 0.0001) among patients reaching PASDAS remission and LDA than those in HDA (Fig. 4). Similarly, higher LSM changes in these PROs were reported by patients in PASDAS remission and LDA versus those in HDA at weeks 52 and 104 (Fig. 4).

**Fig. 5:**
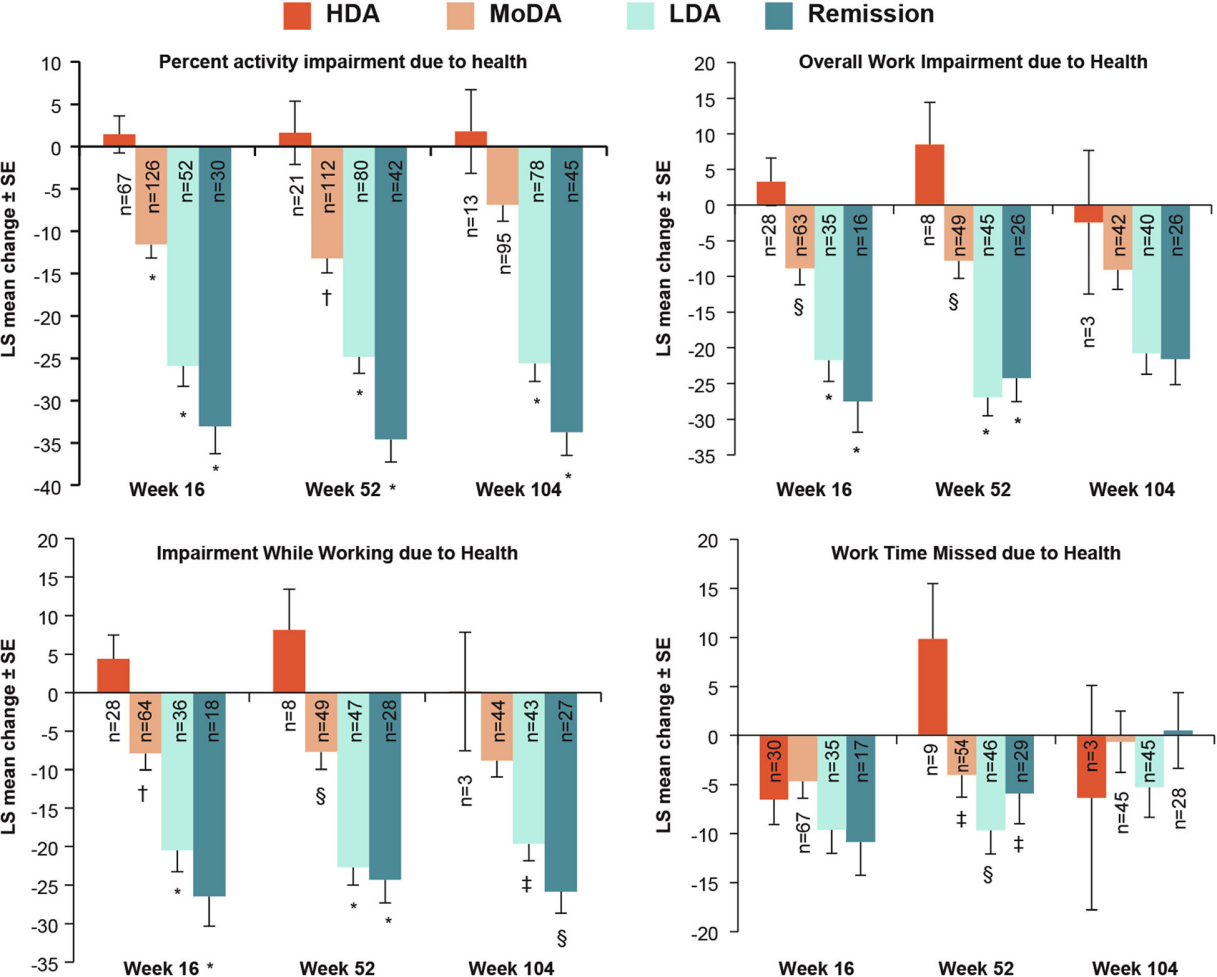
Mean change in work productivity and activity impairment outcome measures by Psoriatic Arthritis Disease Activity Score (PASDAS) states at weeks 16, 52 and 104. Data are from mixed model for repeated measures analysis. *P < 0.0001; ^†^*P* < 0.001; ^§^*P* < 0.01 and ^‡^*P* < 0.05 versus HDA. *n* = number of patients with measurements at both baseline and post-baseline visits; *N* = number of patients in each group of the specified analysis set. *HDA* High disease activity, *LDA* Low disease activity, *LS* Least squares, *MoDA* Moderate disease activity. Week 16: *N* = 32 (remission), *N* = 53 (LDA), *N* = 128 (MoDA) and *N* = 69 (HDA); week 52: *N* = 44 (remission), *N* = 81 (LDA), *N* = 112 (MoDA) and *N* = 21 (HDA); week 104: *N* = 47 (remission), *N* = 81 (LDA), *N* = 95 (MoDA) and *N* = 13 (HDA). Analytic data were pooled across treatment arms

As shown in Fig. 5, a decrease (*P* < 0.0001) from baseline to week 16 in the percentage of activity impairment due to health, overall work impairment due to health, and impairment while working due to health were reported by patients in PASDAS remission and LDA compared with those in HDA. Similar reductions in percentage of activity impairment due to health (*P* < 0.0001) and impairment while working due to health (*P* < 0.05) were reported by patients in PASDAS remission and LDA than HDA at weeks 52 and 104; percentage of overall work impairment due to health was reduced (*P* < 0.0001) at week 52. The percentage of work time missed due to health decreased (*P* < 0.01) from baseline to week 52 among patients in remission and LDA compared with HDA at week 52.

## Discussion

PASDAS is a novel composite index which assesses multiple facets of PsA, including tender and swollen joints, dactylitis, enthesitis, and HRQoL, and offers both a treatment target and a disease activity state assessment across important clinical domains with validated cut points. PASDAS distinguishes treatment effect, performs better than traditional joint-only indices, and could be used as a treatment target in RCTs and longitudinal observational studies in PsA. There are limited data available on PASDAS in two TNFi clinical trials and in two ixekizumab trials. A study on a golimumab data set (GO-REVEAL trial) showed that PASDAS was able to distinguish treatment effect, having larger effect size at week 24 [20]. Another study using data on certolizumab pegol (RAPID-PsA trial) also showed that PASDAS demonstrated good responsiveness and discriminative ability at week 12, supporting further exploration of its use in PsA clinical trials [21]. Assessment of PASDAS-defined LDA and very low disease activity (VLDA) was also reported with ixekizumab data from SPIRIT-P1 and SPIRIT-P2 trials [22] and showed that the proportions of patients achieving PASDAS LDA and PASDAS VLDA were greater with ixekizumab than with placebo [22], further confirming the validity of PASDAS as a measure that can differentiate treatment effect. Therefore, in the present study, the ability of secukinumab to reach PASDAS-related remission or LDA was evaluated using data from the FUTURE 2 trial. To the best of our knowledge FUTURE 2 is the first trial to report detailed PASDAS-based analysis in a mixed TNFi-naïve and TNFi-experienced PsA population treated with an IL-17A inhibitor.

Sustained clinical benefit with secukinumab was previously demonstrated through 104 weeks in the treatment of moderate to severe PsA in the phase III FUTURE 2 trial [15, 16], which included the more stringent clinical end points such as ACR70 responses, PASI 90 response, resolution of enthesitis and dactylitis, and high retention rate as a surrogate marker [15, 16, 23]. Results of this post hoc analysis using PASDAS scores showed that higher proportions of patients treated with secukinumab 300 and 150 mg achieved PASDAS remission or LDA at week 16 than those who received placebo in the overall population at a group level with responses sustained through week 104. Shift analysis of PASDAS states from weeks 16 to 52 and from weeks 16 to 104 confirmed that at an individual level, a majority of patients meeting either PASDAS remission or LDA either maintained or improved their status over time. These data illustrate that secukinumab can meet more stringent treatment goals in line with EULAR and GRAPPA recommendations [6, 7]. Results of this post hoc analysis complement and extend previous reports from the FUTURE 2 trial which have shown that patients treated with secukinumab achieved and sustained remission or LDA defined by other composite indices such as MDA/VLDA and DAPSA up to 2 years [24, 25]. At week 16, in the overall population, the proportions of patients treated with secukinumab 300/150 mg achieving remission were 14%/10% (DAPSA-REM) and 8%/6% (VLDA), respectively, and in those achieving LDA the proportions were 42%/44% (DAPSA REM/LDA) and 28%/23% (MDA) [24, 25], respectively. These results were sustained through week 104.

Secukinumab (300 and 150 mg)-treated patients achieving remission and LDA had improved median scores across all PASDAS core components relating to physician and patient global VAS, SF-36 PCS, SJC 66, TJC 68, dactylitis and enthesitis in contrast to PASDAS HDA. Among these, physician and patient global VAS, TJC 68, SJC 66, dactylitis and enthesitis were most improved.

In the subgroup analysis by previous TNFi use, the proportion of secukinumab-treated patients achieving remission and LDA at week 16 was higher in both TNFi-naïve and TNFi-experienced patients than in patients receiving placebo, with responses in secukinumab groups sustained or increased at weeks 52 and 104. The proportion of secukinumab-treated patients achieving remission and LDA was generally greater in the TNFi-naïve subgroup than in the TNFi-experienced subgroup through week 104. In a recent cross-sectional analysis of a longitudinal cohort, of 79 patients with PsA receiving their first biologic DMARD (32: etanercept, 24: adalimumab, 18: golimumab, and 5: ustekinumab) for at least 6 months, 12.6% achieved remission (PASDAS ≤ 1.9) and 41.8% were in PASDAS LDA [26]. Our study showed in 128 TNFi-naïve patients treated with secukinumab (300 or 150 mg) that 16–28% of patients achieved remission and 37–38% achieved LDA at week 52.

At week 16, a higher proportion of patients treated with secukinumab achieved PASDAS remission or LDA versus placebo, regardless of time since PsA diagnosis (≤ 2 years versus > 2 years). A numerically higher proportion of secukinumab-treated patients reached PASDAS remission or LDA at weeks 52 and 104 than at week 16, regardless of time since PsA diagnosis. Although PASDAS includes SF-36 PCS as one of its core components, patients achieving PASDAS remission and LDA also reported greater improvements in other PROs (HRQoL, mental health, physical function, fatigue and work productivity) through week 104, confirming that these stringent goals translate into improved patient quality of life and function as well as for society owing to higher workforce productivity.

The PASDAS has certain limitations in that, being a complex composite index, it requires complex mathematical calculations, which are time-consuming, although this has been overcome with an application allowing for an easy calculation. PASDAS thus may be more appropriately used in RCTs [10]. It does not include a measure for axial involvement and patient pain, both important manifestations of PsA. Moreover, PASDAS-based publications on RCT data are sparse to place it in context with other composite indices used in PsA. Another limitation of this study was that patients in HDA were not specifically treated to reach the preferable REM/LDA states. Furthermore, while PASDAS change from baseline was an exploratory end point in the FUTURE 2 study, the cut points related to the different disease activity states were recently validated, and this post hoc analysis was generated following the more recently validated cut-offs. These data will require confirmation in new RCTs. Also, there is a lack of assessment in relation to structural outcomes as per the FUTURE 2 study protocol. Further, while we appreciate the fact that SF-36 PCS is one of the core components of PASDAS and therefore a question of circularity in looking at the relationship of PASDAS states and PROs could be raised, we still think it is relevant to do this analysis and see how PASDAS states translate to patient well-being and thus outcomes reported by them.

## Conclusions

In summary, this post hoc analysis of FUTURE 2 data showed that secukinumab treatment resulted in PASDAS remission or LDA at week 16 with responses sustained or further improved through week 104 at the group and individual levels. Improvement or sustainability of MoDA, LDA and remission states were more frequently achieved with secukinumab 300 mg than 150 mg. PASDAS remission/LDA was associated with significantly greater improvements in HRQoL, physical function, fatigue and work productivity. These results extend the previous findings of maintenance of other stringent clinical efficacy end points, including VLDA and MDA, in the FUTURE 2 trial, demonstrating that secukinumab treatment can result in sustained PASDAS-defined remission or LDA, thus demonstrating the potential utility of PASDAS as an outcome measure in RCTs in PsA.

## Abbreviations

ACR: American College of Rheumatology
CPDAI: Composite Psoriatic Disease Activity Index
CRP: C-reactive protein
DAPSA: Disease Activity Index for Psoriatic Arthritis
DAS: Disease Activity Score
DAS-28 CRP: 28-joint Disease Activity Score based on C-reactive protein
DLQI: Dermatology Life Quality Index
DMARD: Disease-modifying anti-rheumatic drug
EULAR: European League Against Rheumatism
FACIT: Functional Assessment of Chronic Illness Therapy
GRAPPA: Group for Research and Assessment of Psoriasis and Psoriatic Arthritis
HAQ-DI: Health Assessment Questionnaire Disability Index
HDA: High disease activity
HRQoL: Health-related quality of life
IL: Interleukin
IRB: Institutional review board
LDA: Low disease activity
LS: Least squares
MoDA: Moderate disease activity
NSAID: Non-steroidal anti-inflammatory drug
PASDAS: Psoriatic Arthritis Disease Activity Score
PASI: Psoriasis Area Severity Index
PRO: Patient-reported outcome
PsA: Psoriatic arthritis
PsAQoL: Psoriatic Arthritis Quality of Life questionnaire
Q: Quartile
RCT: Randomized controlled trial
REM: Remission
s.c.: Subcutaneous
SF-36 MCS: Short Form Health Survey Mental Component Summary
SF-36 PCS: Short Form Health Survey Physical Component Summary
SJC: Swollen joint count
TJC: Tender joint count
TNF: Tumour necrosis factor
VAS: Visual analogue scale
WPAI: Work Productivity and Activity Impairment questionnaire
